# Genetic Predisposition of Atherosclerotic Cardiovascular Disease in Ancient Human Remains

**DOI:** 10.5334/aogh.4366

**Published:** 2024-01-25

**Authors:** Christina Wurst, Frank Maixner, Alice Paladin, Alexandra Mussauer, Guido Valverde, Jagat Narula, Randall Thompson, Albert Zink

**Affiliations:** 1 Eurac Research –Institute for Mummy Studies, Bozen/Bolzano, Italy; 2 Palaeogenetics Group, Institute of Organismic and Molecular Evolution (iomE), Johannes Gutenberg University of Mainz, Mainz, Germany; 3 Medicine & Cardiology, McGovern Medical School, Houston, Texas, USA; 4 Saint Luke’s Mid America Heart Institute, Kansas City, MO, USA

**Keywords:** Polygenic Risk Score (PRS), ancient DNA (aDNA), mummy, atherosclerosis, atherosclerotic cardiovascular disease (ASCVD), calcified atherosclerotic plaques; targeted enrichment capture

## Abstract

**Background:**

Several computed tomographic studies have shown the presence of atherosclerosis in ancient human remains. However, while it is important to understand the development of atherosclerotic cardiovascular disease (ASCVD), genetic data concerning the prevalence of the disease-associated single nucleotide polymorphisms (SNPs) in our ancestors are scarce.

**Objective:**

For a better understanding of the role of genetics in the evolution of ASCVD, we applied an enrichment capture sequencing approach to mummified human remains from different geographic regions and time periods.

**Methods:**

Twenty-two mummified individuals were analyzed for their genetic predisposition of ASCVD. Next-generation sequencing methods were applied to ancient DNA (aDNA) samples, including a novel enrichment approach specifically designed to capture SNPs associated with ASCVD in genome-wide association studies of modern humans.

**Findings:**

Five out of 22 ancient individuals passed all filter steps for calculating a weighted polygenic risk score (PRS) based on 87 SNPs in 56 genes. PRSs were correlated to scores obtained from contemporary people from around the world and cover their complete range. The genetic results of the ancient individuals reflect their phenotypic results, given that the only two mummies showing calcified atherosclerotic arterial plaques on computed tomography scans are the ones exhibiting the highest calculated PRSs.

**Conclusions:**

These data show that alleles associated with ASCVD have been widespread for at least 5,000 years. Despite some limitations due to the nature of aDNA, our approach has the potential to lead to a better understanding of the interaction between environmental and genetic influences on the development of ASCVD.

## Introduction

Atherosclerotic cardiovascular disease (ASCVD) is a multifactorial disorder and the main cause of death worldwide [1]. Inflammatory processes lead to a change in the intima of the blood vessels, mainly due to the accumulation of lipids developing into a plaque. This eventually leads to reduced blood flow or thrombus formation, which can result in a heart attack or stroke [2].

Beyond classic cardiovascular risk factors such as obesity, hyperlipidemia, hypertension, and smoking, epidemiologists estimate that 50% of the risk of atherosclerosis is hereditary [3,4]. Because of this, the analysis of genetic predisposition for atherogenesis has become a major research focus in recent years.

In 2007, four independent research groups started to use genome-wide association studies (GWASs) to find new loci to better understand the genetic component in the development of the disease [5,6,7,8]. Thus far, more than 1,790 loci associated with ASCVD have been identified. However, most of the single nucleotide polymorphisms (SNPs) have low impact, and predisposition for ASCVD comes from accumulation of inherited disease alleles weighted by their effect size, which can be expressed by a weighted polygenic risk score (PRS) [9]. In addition, etiologic mechanisms are still not completely understood, and only about half of the known SNPs can be assigned to a pathophysiological pathway [4]. Research is ongoing, and just recently, 95 new loci were discovered [10].

During the past 15 years, there have been an increasing number of studies finding evidence for ASCVD in ancient human remains using non-invasive methods such as computed tomography (CT). Mummies from North and South America, Greenland, Europe, North Africa, and East Asia show that the disease affected individuals with different lifestyles and environmental conditions, and that it has been with us for over 5,000 years–much longer than previously widely believed [11,12,13,14,15,16,17].

The question remains whether our ancestors suffered from the same genetic risk of developing ASCVD as we do today. Only two mummies–“Ötzi” the Iceman, an Italian glacier mummy from 3300 BC (hereafter referred to as *the Iceman*), and a Korean mummy from the 17^th^ century–have been shown to have particular SNPs associated with coronary artery disease (CAD) linked to the physical occurrence of calcified plaques in arteries [18,19,20]. One reason for the scarcity of data is that a link between the genotype and phenotype can only be achieved in mummified individuals. In mummies, the soft tissue is often sufficiently preserved to examine organs or arteries for diseases that have left no traces on the skeleton [21]. Another reason is that most available genotypes from ancient individuals are pseudo-haploid as a consequence of low genotyping coverage [22]. Ancient DNA (aDNA) is highly fragmented and prone to contamination. Therefore, the endogenous DNA content of a sample is often below 1% [23], which results in a low sequencing depth for human reads.

In this study, we investigate the genetic burden of ASCVD in 22 ancient individuals, with the aim of expanding the genetic data associated with ASCVD in ancient human remains. Therefore, a targeted enrichment capture was designed to analyze selected SNPs in the DNA of ancient human remains from different time periods and provenances. A weighted PRS for the ancient individuals was calculated and compared to the genetic predisposition of modern individuals.

## Materials and Methods

### Sample material

**Figure 1:**
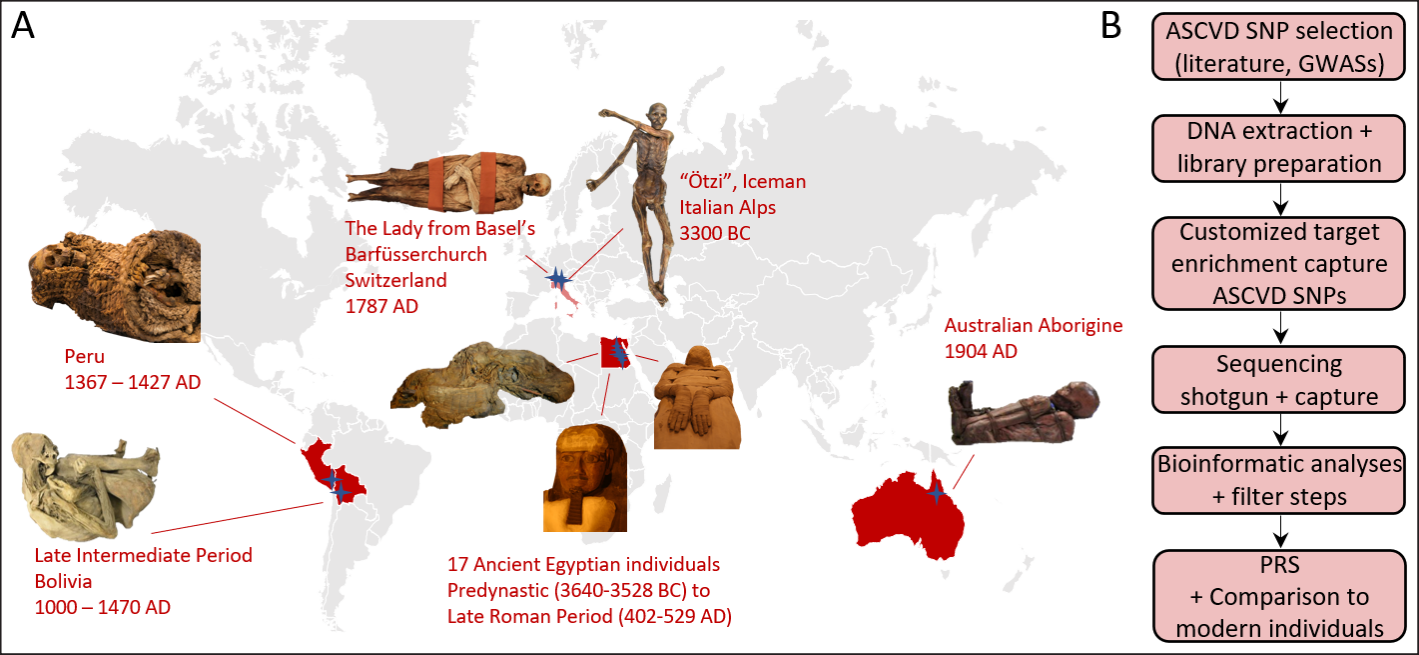
**Mummified individuals and workflow of this study.** (A) Human remains from four different continents were analyzed covering a time span of about 5,500 years of human history. Stars represent the finding sites. (B) The workflow containing in silico, laboratory, and bioinformatic analyses with the final evaluation of polygenic risk scores (PRS).

The ancient sample material used in this study derived from mummified and skeletonized human remains from various geographic areas and time periods, including 17 individuals from Ancient Egypt (3640 BC–655 AD), one individual from Bolivia (1000–1470 AD), one individual from Peru (1367–1427 AD), one church mummy from Switzerland (1787 AD), and one Australian aboriginal mummy (1904 AD). In addition, the genetic data of the new genome from the Iceman (3350–3120 BC) has been included in the analyses [24] (Figure 1A; Supplementary Table S1).

For comparison with modern humans, genetic data of 2,504 unrelated individuals from 26 populations analyzed in the final phase three of the 1,000 Genomes Project were used [25].

### Sample processing

Depending on the accessibility to the human remains, and in order to minimize the necessary destruction to a minimum, samples were taken from different bone parts (see Supplementary Table S2). The analyses were performed in the ancient DNA Laboratory of the Eurac Research Institute for Mummy Studies–a special DNA Laboratory in Bolzano (Italy) dedicated to the work with ancient biomolecules. To avoid modern or ancient cross-contamination, standards for working with ancient material in DNA analyses were followed [26]. DNA was extracted from the samples using different methods. Further information can be found in Supplementary Table S2. Double-stranded DNA-libraries were prepared from 25 µl of DNA extracts following the protocol of Meyer and Kircher [27]. Quantity and quality controls of the DNA-libraries were performed using fluorescence-based methods (Quantus [Promega]; Bioanalyzer [Agilent]). Subsequently, the libraries were shotgun sequenced on an Illumina HiSeq instrument (101 bp read length PE) to obtain a comparison for the capture performance.

### ASCVD capture

To analyze the genetic predisposition of ASCVD in ancient human remains, a DNA enrichment capture based on the genome-wide association studies (GWAS) and large-scale association analyses available at that time was designed in early 2016. A total of 163 SNPs fulfilling certain criteria (e.g., p-value, occurrence in multiple studies) were selected to create a customized in-solution hybridization capture based on 80 mer-RNA baits (for details to the capture design refer to Supplementary S1_SNP catalogue, S2_Capture design, Table S4, Figure S1). The capture has been performed according to the recommendations of the Daicel Arbor Biosciences manual (v3.02) for aDNA. Modifications of the protocol, like different hybridization temperatures and a double or single round of capture applications were tested. Captured DNA libraries were sequenced with different Illumina HiSeq instruments (see Supplementary Table S3).

### Bioinformatic analyses

The forward and the reverse read of raw data were merged with a minimum length of merged sequences of 25 bp and a minimum number of overlapping bases of 25 bp using PEAR (v.0.9.10) [28]. With the QualityFilterFastQ.py script [29] reads with five bases below the quality threshold of 15 were removed. BWA (v.0.7.16a) [30] with a seed length of 1,000 was applied to align the sequencing reads to the human reference genome (build hg19) and the mitochondrial reference genome (rCRS) [31], respectively. The files were converted into BAM files using SAMtools (v.1.9) [32], removing reads with a mapping quality below 25. Read duplicates were removed using DeDup (v.0.11.3) [33]. Schmutzi [34], an iterative approach to estimate human contamination in ancient DNA datasets based on the mitochondrial reads was applied. For a further authentication of aDNA, MapDamage2 (v.2.0.8) [35] was applied to retrieve deamination patterns typically for aDNA (Supplementary Figure S2). With the same tool, a new BAM file was constructed in which mutations sequenced most likely due to damage to ancient DNA received a downscaled quality value. The rescaled BAM files were converted into VCF files using BCFtools (v.1.17) [32], including only the loci of the 163 ASCVD SNPs. For SNPs that are deletions, the surrounding bp were checked to make sure this position was a real deletion. If the analyzed locus was a stretch or short tandem repeat (STR), more bps were analyzed until the end of the repetition. For the final evaluation, only SNPs with a minimal coverage of five were considered.

For the modern individuals, the targeted ASCVD SNPs were extracted from the single chromosome files (zipped VCF) using tabix from SAMtools [32]. Afterwards, the achieved TSV files were combined into one VCF file using BCFtools concat [32] containing all analyzed ASCVD loci of the 2,504 unrelated individuals from final phase 3 of the 1,000 Genomes Project [25]. PRS were calculated in the same way as for the ancient individuals. A density curve for the PRSs of modern individuals was plotted with R using ggplot2. The PRSs of the ancient individuals were plotted on top of the density curve of the modern individuals.

### Comparison shotgun vs. capture data (Normalization)

To see if the capture approach really increased the number of captured targets, a normalization of the captured datasets was performed based on the read number of shotgun data of each analyzed individual. Therefore, in a first step, Seqkit (v.0.8.1) (rmdup) [36] was used to remove duplicates by sequences of the unaligned (but merged and quality filtered) FASTQ files of the shotgun and capture datasets. Based on the number of remaining reads in the shotgun dataset, the capture dataset was down-sampled using Seqtk (v.1.3) [37]. As described above, the datasets were aligned to the human reference genome hg19 were converted into BAM files, and were rescaled using MapDamage2. The number of reads covering the 163 ASCVD SNPs were counted using SAMtools [32].

### Calculating polygenic risk scores (PRS)

**Figure 2:**
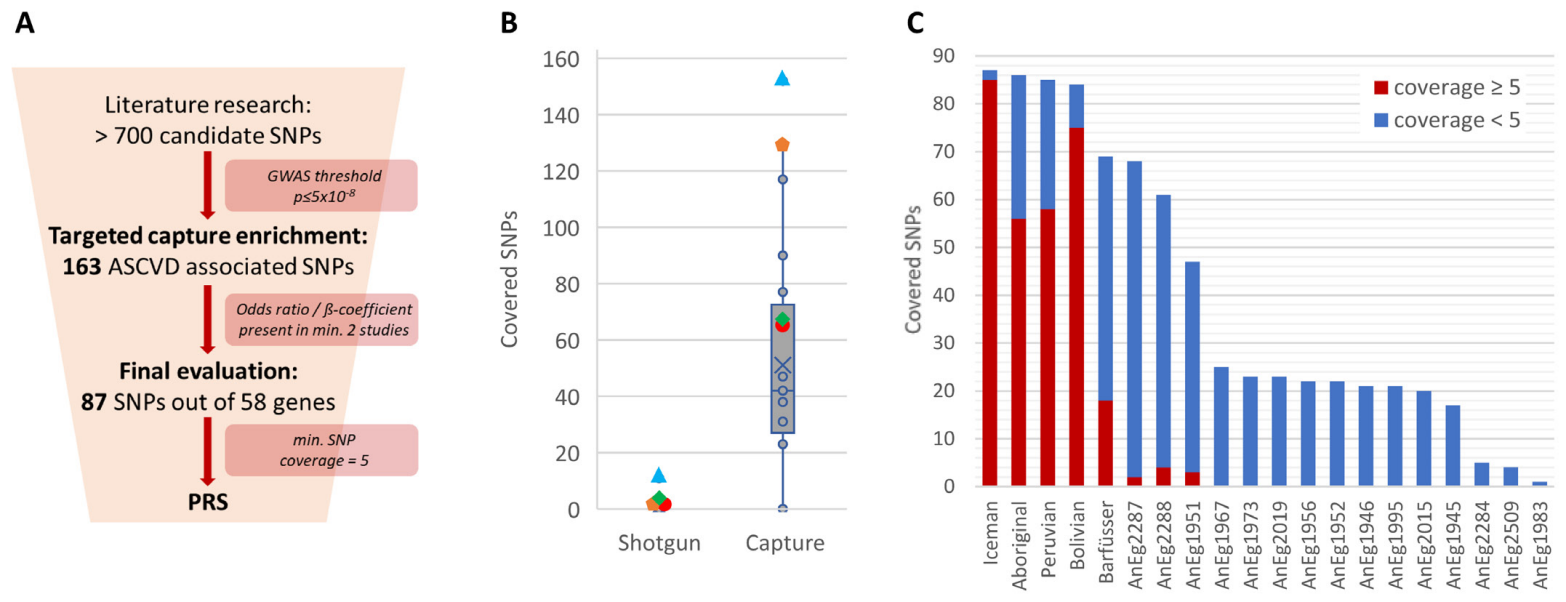
**Study design and SNP coverage. (A)** Filter steps to receive the final number of ASCVD-associated SNPs for the calculation of the weighted risk score. (B) Comparison between achieved numbers of the 163 targeted SNPs of shotgun datasets and the normalized captured datasets. Colored symbols indicate the four individuals with a few covered target SNPs already after shotgun sequencing. (C) Sequenced SNPs of the final 87 SNP collection were divided in a coverage below 5 (blue) and 5 and above (red). The latter are used for the calculation of PRSs.

**Figure 3:**
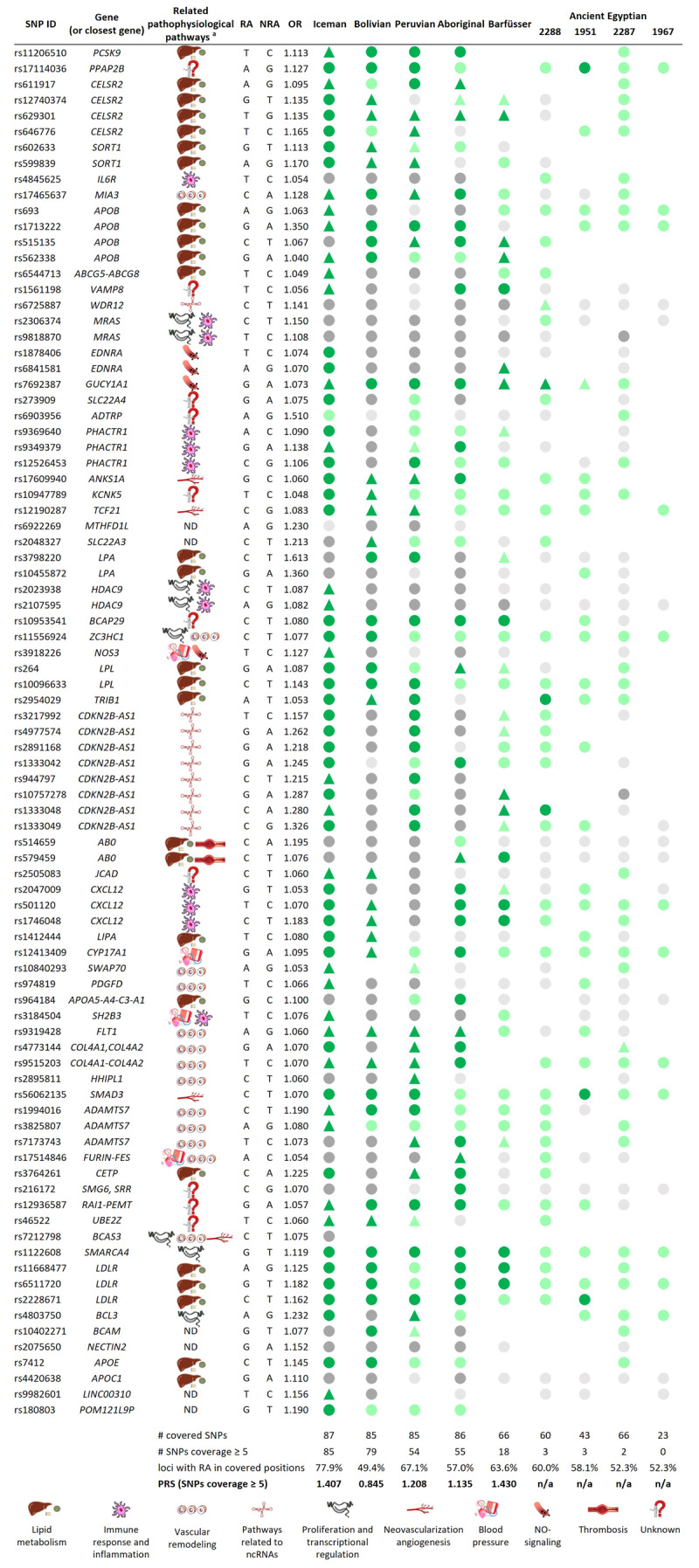
**Overview of the 87 final SNPs and calculated PRSs.** RA = risk allele, NRA = non-risk allele, circles = homozygote state, triangle = heterozygote state, green = RA is present, grey = RA is not present, dark color = coverage ≥ 5; light color = coverage < 5; empty spot = locus has not been covered, ND = not determined, n/a = not applicable.^a^Related pathophysiological pathways based on [4,38,39].

A weighted risk score was calculated for the final evaluation. An additional reduction of SNPs was necessary (Figure 2A) since the disease-causing alleles, and thus, the direction of the mutations was not described in all published studies. Therefore, all initially selected SNPs were re-evaluated in 2022 using currently available studies. Hence, from each SNP, the respective odds ratio (OR) was collected from the reference only if the SNP achieved the genome-wide significance threshold of the*p*-value (*p* ≤ 5 × 10^–8^). SNPs were only considered when they reached the significance threshold in at least two independent studies. If only the beta coefficient was presented, the OR was calculated out of the beta. The total OR per SNP used as a weight for the PRS (Figure 3) was calculated by the mean of the ORs of the different studies (Supplementary Table S5). For alleles of SNPs leading to a higher level of high-density lipoprotein cholesterol (HDLc), and therefore protective against atherogenesis, the other allele was used for the calculation of the weighted risk score. PRSs were calculated by dividing the sum of the weighted allelic states of the risk allele (0X, 1X, 2X) by the number of SNPs that were covered at least five times. The division by the number of covered SNPs is importantsince often not all targeted loci are covered in ancient DNA studies. The formula is shown below.


\[
PRS = \frac{{\sum\nolimits_i^N {O{R_i}\;*\;allelic\;stat{e_i}} }}{{\# \;covered\;SNPs}}
\]

For the final evaluation, both captured and shotgun datasets of one individual were merged and analyzed.

## Results

In this study, 22 ancient individuals were analyzed for a genetic predisposition to atherosclerosis using a targeted enrichment capture approach. DNA from 21 individuals with endogenous DNA contents between 0.04% and 20.93% was successfully extracted (Supplementary Table S1). For the Iceman, previously published sequencing data were used [24]. The extracted DNA samples were first shotgun-sequenced to create a baseline for investigating the efficiency of the capture of the 163 SNPs included in the capture design.

Figure 2B displays the capture efficiency of the 163 ASCVD SNPs included in the capture design. The number of covered SNPs using the enrichment approach increased significantly compared to the analysis of shotgun datasets (see also Supplementary Figure S3 and Table S6). In four (the Bolivian, the Peruvian, and two ancient Egyptians [2287, 2288]) of the 21 individuals analyzed, some (1 up to 11) of the 163 targeted ASCVD-associated SNPs had already been sequenced by shotgun sequencing. With the enrichment approach, an increase of up to 126-fold in covered SNPs was observed with the same number of sequenced reads (normalization). All other individuals achieved a coverage of at least one of the 163 SNPs after the capture approach (Supplementary Table S4).

For the final evaluation, the reduced SNP catalogue of 87 SNPs out of 58 genes was applied (Figure 2A, 2C, Supplementary Figure S5). After the capture approach, one individual (2516) showed a mitochondrial contamination higher than 5% (Supplementary Table S8). In another individual (1953), none of the final 87 ASCVD SNPs were covered. Therefore, both individuals were excluded from further analyses. In addition, to calculate a PRS, only SNPs with a minimal sequencing depth of 5 were considered. Because it has previously been shown that a PRS calculated from 12 genetic risk variants has only a minor improvement in predicting ASCVD events [3], PRSs were calculated only for individuals with at least 15 covered loci with a sequencing depth greater than four, resulting in a PRS of five ancient individuals. The result is shown in Figure 3. In addition, as several ancient Egyptian individuals also showed the presence of selected targeted disease alleles without meeting the sequencing depth criteria (Figure 2C), the four best Egyptian individuals were included in Figure 3 to indicate the prevalence of these disease-causing SNPs in ancient Egypt, without calculating a PRS.

**Figure 4:**
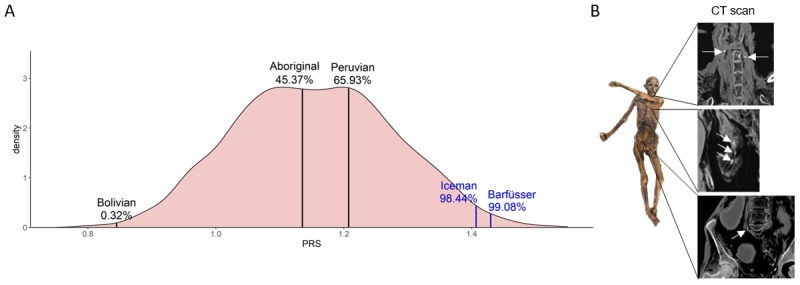
**Comparison of PRSs of modern and ancient individuals. (A)** Density curve of PRSs of 2504 unrelated modern individuals from 26 populations from all over the world. PRSs of ancient individuals are plotted on top of the modern individuals. The percentage displays the number of modern individuals with a lower PRS than the ancient individual. In blue: Individuals with calcified plaques identified on CT scans. (B) In CT scans of the Iceman calcifications were found within the carotid artery, the area of the heart, and the right iliac artery.

We next compared the PRSs of the ancient individuals with the PRSs of modern individuals. The PRSs of the ancient individuals cover the complete range of PRSs of modern individuals (Figure 4A, Supplementary Tabs. S10 and S11). Only 0.32% of modern individuals resulted in a PRSs smaller than the PRS calculated for the Bolivian mummy, while less than 1% of modern individuals showed a higher PRS than the Barfüsser mummy. Thus, our genetic results are consistent with the CT-based analysis of atherosclerosis in the mummies. Of the five mummies with calculated PRSs, calcified arterial plaques were detected only in the Iceman and the Barfüsser mummy, the two ancient individuals with the highest calculated PRS [40,41,42] (Figure 4B).

Raw data generated for this study are publicly available on the European Nucleotide Archive (ENA) under accession number PRJEB62880.

## Discussion

This study combines the genotype and phenotype of ASCVD of several mummies from different parts of the world and from different time periods. Included is the mummy of the 5,300-year-old Iceman, the most ancient published case of proven calcified atherosclerotic arterial plaques. The Iceman was previously diagnosed with a genetic predisposition for cardiovascular diseases based on only a few SNPs [18,19]. These results were confirmed and extended in the present investigation. Together with the additional mummies presented in this study, it is clear that many of the disease-causing alleles were widely distributed in ancient times.

Previous studies have begun to explore the evolution of various complex traits by using ancient DNA [22,43]. In contrast to our investigation, these studies were based on pseudo-haploid genomes in which only one of the two alleles at each locus was randomly selected for evaluation. In these cases, the true allele frequency is obscured, and the absence of a mutation can be artificially created. Recently, Kuijpers and colleagues used this method to show that HDL levels, an antagonist of atherogenesis, decreased consistently in European populations after the Neolithic Revolution [22]. However, in contrast to our study, they were not able to compare their genetic results with real phenotypes and the PRSs were calculated only for a haploid state. For the analysis of complex diseases such as ASCVD, it is important to understand the state of both alleles, inherited by the mother and the father. Therefore, the enrichment approach presented here can help to provide a measure of disease liability of our ancestors by providing the biallelic state of a disease SNP. Another advantage of such a specific enrichment is that only a few ancient whole-genome datasets are available and, especially in the analysis of aDNA, employing an enrichment approach can provide enormous cost savings compared to a whole-genome sequencing approach [44]. So far, more than 70% of published genome-wide ancient human DNA datasets were generated with the 1240 K reagents, a feature included in the latest commercially available genome-wide enrichment assays (Daicel Arbor Biosciences, Twist Bioscience) [44]. However, not all the SNPs used to calculate the PRS for ASCVD of this study are included in those commercially available enrichment assays, as they were initially designed for the study of variation among modern human populations. Even though there are still some technical issues, such as a variable efficacy in different ancient specimens and the coverage of targeted SNPs from the capture design, the enrichment approach presented here is superior to shotgun sequencing. It increases the coverage of the targeted SNPs as well as the sequencing depth for individual SNPs, while reducing the required total sequencing depth.

Notably, only five of the initial 22 samples generated sufficient sequencing data to calculate a reasonable PRS, and this may be due to several reasons. The latest commercial whole-genome assays require a DNA input of at least 1000 ng DNA, although there have been aDNA studies with endogenous contents below 0.4% where 500 ng DNA is sufficient to obtain reasonably good capture results [45]. This shows that both the endogenous content of a DNA sample and the DNA concentration used to perform an enrichment are important for a successful outcome. An improvement of the presented capture results would certainly be achieved by starting with an increased DNA concentration. However, a major bottleneck in many aDNA studies is not only poor sample preservation causing highly fragmented DNA, but also the amount of available sample material. For example, in case number 2284, a sample from an Ancient Egyptian individual, the endogenous content of the extract is above 1%, but only 20 ng of DNA were used to accomplish the enrichment (see Supplementary Tabs. S1 and S3). Therefore, it is extremely difficult to cover all targeted SNPs, much less to receive a sequencing depth of at least five. The repetitive elements within the human genome also present a challenge. The high proportion of repetitive elements (approximately 66%) [46] can lead to an ambiguous positioning of short sequences that are typical for aDNA fragments. These sequences must be removed bioinformatically, as it is impossible to identify their correct position, which can lead to a loss of single SNPs. Although no PRSs could be calculated for the Egyptian individuals (due to the reasons mentioned above), a variety of risk alleles for ASCVD were found, dating back to the First Intermediate Period (Ind. 1967: cal BC 2131–1903).

For the final evaluation of this study, 87 SNPs were selected, recognizing that the predictive accuracy of PRSs increases when employing a higher number of SNPs. However, for some of the 163 initially selected ASCVD SNPs included in the capture design, it was not clear which is the effect allele and which is the non-effect allele. If assumptions were made and turned out to be incorrect, the effect would be calculated in the wrong direction when computing the PRS. Therefore, an additional reduction of SNPs was necessary before calculating the PRSs. This step was also used to reduce a possible bias when the same approach is used to calculate PRSs for individuals of different origins. During the design and implementation of the enrichment approach, we had to consider that most GWASs are based on Europeans, which may lead to a bias in the calculation of PRSs when the same module is applied to non-European populations [22,47]. In 2015, when the SNPs for the capture design were collected, only about a fifth of available genome-wide datasets were generated on people with non-European ancestry [48]. Thus, we included more recent GWASs based on more non-European origins in the reduction for the final SNP catalogue and weighting of these SNPs (Supplementary Table S5).

In addition, we are not only analyzing individuals of different geographical origins, but the populations also differ in terms of time. Therefore, we would also have to consider temporal changes in the genetic composition of populations. Yet, the currently available ancient genomes fall significantly short of the required quantity to build an independent validation data set.

Even in modern populations, there exists no method that really solves the problem of transferability of PRSs based on GWASs from one population to another [49]. We did, however, investigate the distribution of the calculated PRSs in individuals of the five individual superpopulations (based on the classification of the 1,000 Genomes Project) (Supplementary Figure S6). With this approach, similar shapes of the density curves for all superpopulations were achieved with one exception. As already known from previous studies, we have seen that our method is not applicable to individuals from sub-Saharan Africa, since the genetic distance is too high [49].

There are two additional issues linked to GWASs that are worth mentioning. Firstly, GWASs mainly detect common variants, often with a high minor allele frequency and only a small effect size [4,50]. It has been noted that over 50% of the ASCVD-associated SNPs occur in over 50% of the European population [3]. Secondly, the mere discovery of associated SNPs reveals nothing about their mode of action. Over 80% of disease-associated variants discovered with GWASs are within non-coding regions. Therefore, it is difficult to identify specific genes, and, so far, their role in atherogenesis can be ascertained for only about half of the SNPs [3,4]. This problem is the reason why there is no linked pathophysiological pathway for many SNPs in this study (Figure 3). Furthermore, at the time of SNP selection, variants related to lipid metabolism were primarily the ones identified. Whether the fact that European ancestry predominating in GWASs plays a role remains to be investigated, as does the question of whether there are other risk alleles for non-Europeans that were not considered in this study.

Nevertheless, after the final evaluation of our data, the two individuals with the highest PRS were the only two individuals showing arterial calcifications on the CT scans. Remarkably, these are also the only two Europeans in this study. In contrast, the ancient Bolivian showed the lowest PRS and no atherosclerotic plaques. This could indicate that atherosclerosis was less common in ancient Bolivians, as very low rates of atherosclerosis have been demonstrated in a contemporary Bolivian lowland forager-horticulturalist population that has maintained a pre-industrial lifestyle, the Tsimane. This population has a five-fold lower prevalence of coronary calcifications than industrialized populations [51].

The assessment of arterial calcifications in mummies is very much dependent on the state of preservation. In a mummified body, it can be difficult to distinguish between calcifications of arteries and calcifications of another origin, due to shrinkage and movement of the original position of organs and arteries during the process of mummification [21]. This could be why no arterial calcifications were discovered in the two individuals of the intermediate risk group–arteries may have been affected but did not survive the ages. In particular, the Peruvian individual had only limited soft tissue preservation, making it difficult to identify any atherosclerotic arterial plaques. There is also a strong correlation between the age of an individual and the development of atherosclerotic calcifications. Some of the mummified individuals with a genetic tendency for atherosclerosis may have simply not lived long enough to develop it.

All in all, there is always an interplay between an individual’s genetic composition and lifestyle circumstances that leads to the development of ASCVD plaques. Even if many traditional cardiovascular risk factors such as smoking and sedentary activity levels were less prevalent in the past, it is challenging to reconstruct a complete lifestyle of an individual and find environmental conditions that contribute to the pathophysiology. For example, the last meal of the Iceman indicated a lipid-rich diet [52] and he is thought to have been constantly exposed to smoke from fireplaces. At the same time, he had a high energy consumption, and it is not known whether wood fire smoke has a similar impact on atherosclerotic calcifications as tobacco smoke [19]. Further studies to determine the interaction of different life conditions of the past and the development of ASCVD-associated SNPs are needed.

Although we have studied only a limited number of mummies, we have noted indications for a relation between the calculated PRSs and the presence of atherosclerotic plaques in them. It is still difficult to reconstruct the lifestyle and thereby the presence of traditional risk factors in ancient populations. Thus, the PRS has the potential to serve as a proxy for estimating the risk for developing ASCVD in ancient humans. Importantly, a PRS is not age-dependent and cannot be influenced by environmental factors [3]. With the investigation presented here, we developed a tool that can be used to conduct further genetic studies of ancient humans of different geographic origins and time periods to gain a better understanding of the development of ASCVD.

## Additional Files

The additional files for this article can be found as follows:

10.5334/aogh.4366.s1 Supplementary File 1. Supplementary Methods Text S1–S2 and Supplementary Figure S1–S6.

10.5334/aogh.4366.s2 Supplementary File 2. Supplementary Table S1–S11.
