# Supplementary material for: Genetic Predisposition of Atherosclerotic Cardiovascular Disease in Ancient Human Remains
Source: Ann Glob Health. 2024 Jan 25;90(1):6. doi: 10.5334/aogh.4366 (PMC10809863; doi:10.5334/aogh.4366)
Supplement: Supplementary File 1. — Supplementary Methods Text S1–S2 and Supplementary Figure S1–S6. [file agh-90-1-4366-s1.pdf]

S1 SNP catalogue

For the decision of which SNPs should be included in the capture design, a literature research for SNPs associated with cardiovascular diseases was performed in 2015. More than 700 candidate SNPs were found. SNPs were considered for the capture design when they were present with the genome-wide significance threshold of $p \leq 5 \times 10^{-8}$. Eleven SNPs were included in the SNP catalogue that were associated with ASCVD in East Asians (N=10) or in American natives (N=1) but not in Europeans, to analyze their occurrence in ancient samples of various geographic origins. This resulted in an ASCVD associated SNP catalogue of 163 SNPs. Further nuclear markers were added to the capture design to get a broader knowledge about the sampled individual itself. Twenty six SNPs were added to predict the eye, hair, and skin color [1,2]; seven SNPs associated with the blood group (6 SNPs for the AB0 system + 1 SNP secretor / non-secretor) [3,4]; five SNPs for lactase persistence (2 SNPs: Europeans + 3 SNPs Sub-Saharan Africans) [5]; two SNPs of the *UMOD* gene which are associated with chronic kidney disease (CKD) risk and urinary tract infections [6]; and 454 Y-chromosomal SNPs to analyze the Y-haplogroup of male individuals. The selection of the Y-chromosomal SNPs was based on a study of Ralf and colleagues [7] with 530 Y-chromosomal SNPs (based on ISOGG 2013). A further refinement of these SNPs was performed manually (based on ISOGG Y-DNA Haplogroup Tree 2016, Version 11.92). Two SNPs were kept per main-haplogroup and one SNP for deeper subclades. Furthermore, the depth of subclades was evaluated by geographical relevance. In the end, 454 Y-chromosomal SNPs were added to the SNP catalogue. The list of all SNPs included in the enrichment can be found in Supplementary table S4.

S2 Capture design

A customized in-solution hybridization capture was designed in cooperation with Daicel Arbor Bioscience. 80-mer RNA baits were designed for regions of 251 bp covering the targeted SNP in the center of this regions (based on hg19). In cases where two SNPs were within the range of 125 bp, the two areas were combined for the bait design. For the main targets, the ASCVD SNPs, baits were

designed with a 10 x tiling (8 bp spacing) in 150 regions, for baits of the additional SNPs a 5 x tiling (16 bp spacing) was used for 373 regions, which resulted in 8817 unfiltered bait candidates. To achieve an optimal compromise between capturing the important targets while having good capture efficiency, those candidates were filtered based on a BLAST-filter step and a RepeatMasking-filter step.

For each bait candidate the blast hits (against the hg19 genome) with their melting temperature (Tm) were counted in six different temperature bins (40-60°C, 60-62.5°C, 62.5-65°C, 65-67.5°C, 67.5-70°C, and above 70°C). Bait candidates were accepted based on the BLAST-filter step when they fulfilled at least one of the following five criteria: I) no blast hit with a Tm above 60°C, II) no more than 2 hits at 62.5 – 65°C, or 10 hits in the same interval and at least one neighbor candidate being rejected, III) no more than 2 hits at 65 - 67.5°C and 10 hits at 62.5 - 65°C and two neighbor candidates on at least one side being rejected, IV) no more than a single hit at or above 70°C and no more than 1 hit at 65 - 67.5°C and 2 hits at 62.5 - 65°C and two neighbor candidates on at least one side being rejected, V) no more than 10 hits in the bin of 62.5 - 65°C and no more than 4 hits in the bins above 65°C while they were not surrounded by two consecutive selected baits on each side. This resulted in 508 of the 523 regions that were covered with at least one bait. The bait candidates were further filtered based on the RepeatMasking-filter step. This step identifies repetitive elements (e.g., LINEs, SINEs), simple repeats (e.g., STRs), and low complexity DNA sequences, which can lead to the capture of off-target sequences. For the final capture design, the following bait candidates were taken: I) all non Y-chromosomal baits that passed the above mentioned BLAST-filter, regardless of the RepeatMasking-filter, II) all Y-DNA baits failing the BLAST-filter were removed, III) all Y-DNA baits passing the BLAST-filter step but having a RepeatMasking value above 60% were removed, IV) for SNPs where all baits were removed based on the previous steps (except for Y-DNA SNPs), at least 4 baits with the fewest overall hits were included (if possible baits covering the actual target SNP(s) were chosen), V) if baits of a specific SNP were only covered on the end of one side of the 251 bp region (e.g., only baits starting from 104 to 172 bp passed

the filters) then one bait from the other side was added as well (e.g., a bait starting between 48 and 64 bp was added), VI) at least one SNP of each Y-DNA region was added based on the fewest overall hits and the RepeatMasking value. The bait of the 251 bp region that started at 80 bp was preferably chosen. If it was a combined region, further baits were included to cover each SNP-region with at least 1 bait. In total, 7166 unique RNA baits were included in the capture design.

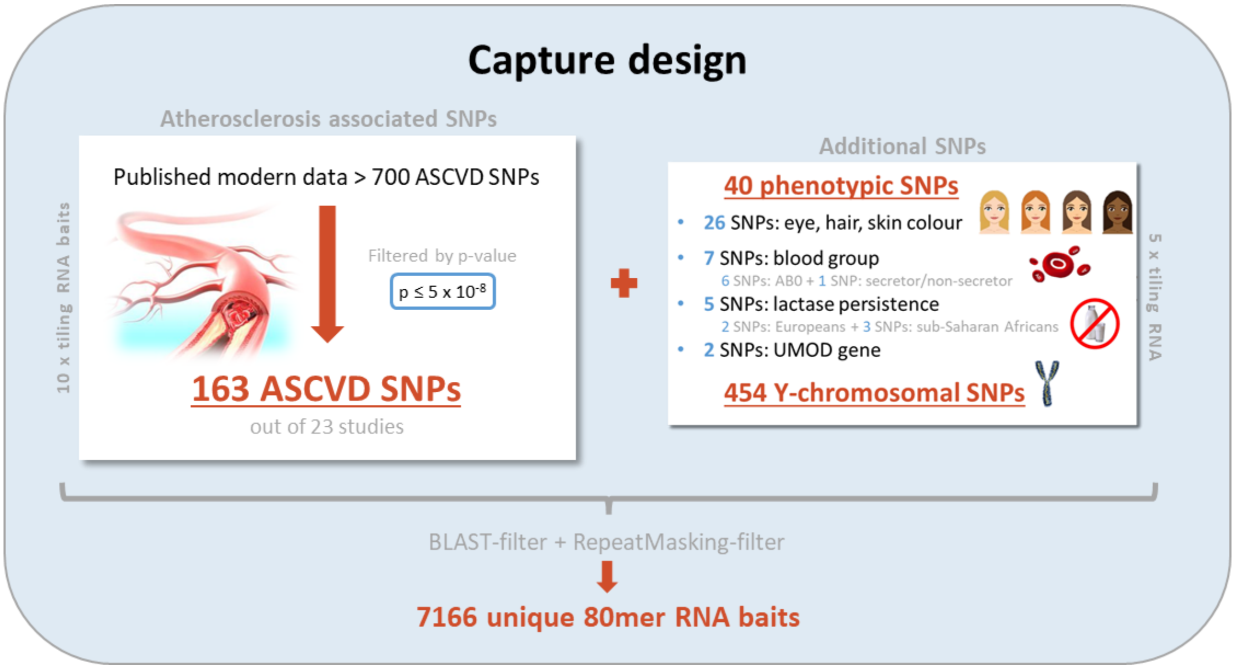

Figure S1: Capture design

A)

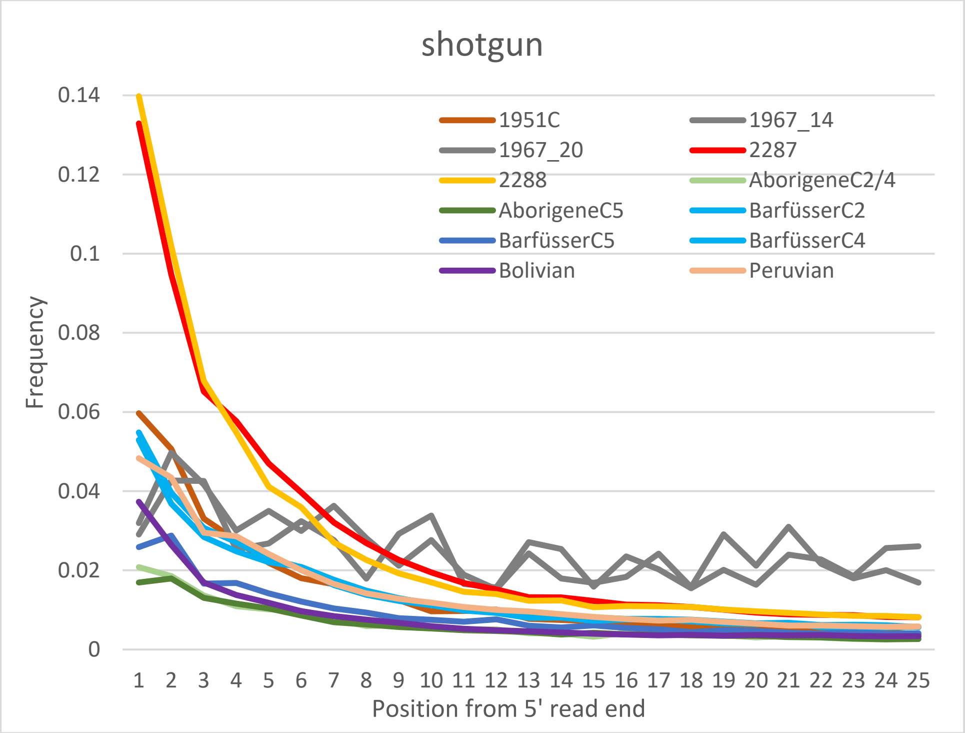
shotgun
0.14
0.12
0.1
0.08
0.06
0.04
0.02
0
Frequency
1951C
1967_14
1967_20
2287
2288
AborigeneC2/4
AborigeneC5
BarfüsserC2
BarfüsserC5
BarfüsserC4
Bolivian
Peruvian
1 2 3 4 5 6 7 8 9 10 11 12 13 14 15 16 17 18 19 20 21 22 23 24 25
Position from 5' read end

B)

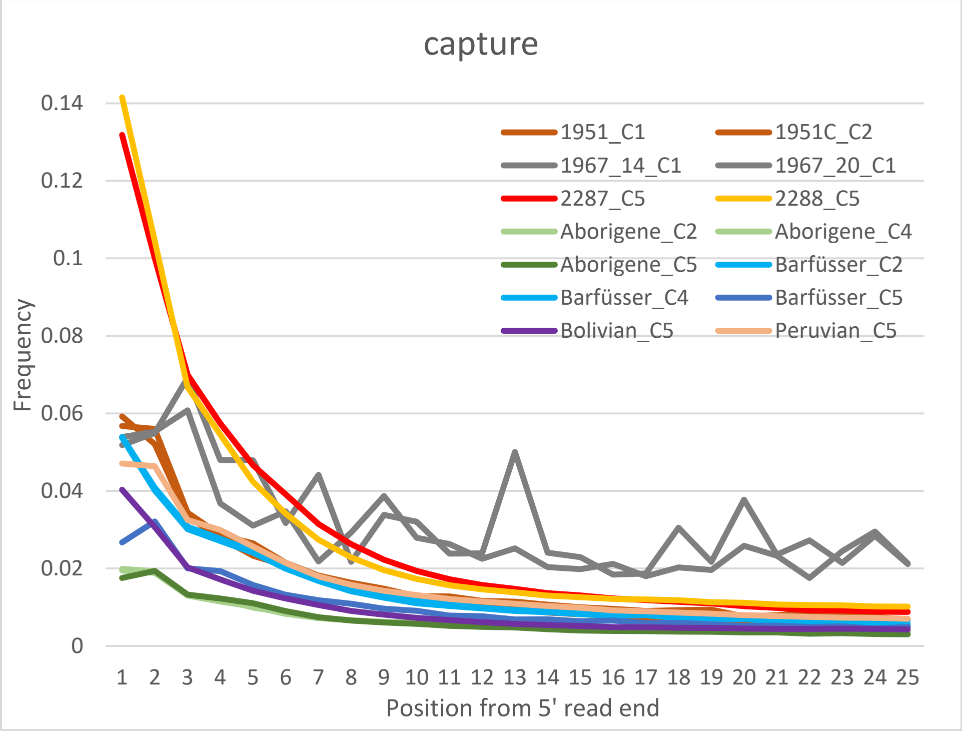
capture
0.14
0.12
0.1
0.08
0.06
0.04
0.02
0
Frequency
1951_C1
1951C_C2
1967_14_C1
1967_20_C1
2287_C5
2288_C5
Aborigene_C2
Aborigene_C4
Aborigene_C5
Barfüsser_C2
Barfüsser_C4
Barfüsser_C5
Bolivian_C5
Peruvian_C5
1 2 3 4 5 6 7 8 9 10 11 12 13 14 15 16 17 18 19 20 21 22 23 24 25
Position from 5' read end

Figure S2: Damage pattern

For aDNA characteristic damage patterns based on a T to C transition of bases from the 5' end of sequencing reads of A) the shotgun datasets and B) the capture datasets, showing no significant difference between shotgun and capture data.

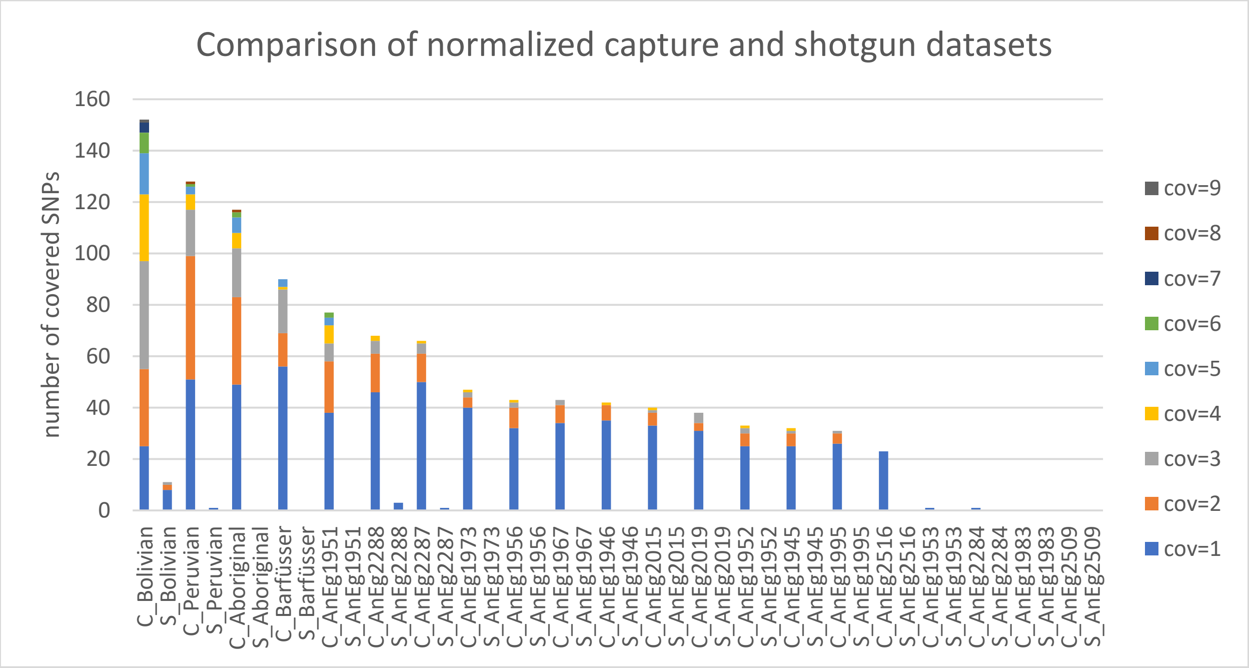

Figure S3: Comparison of the coverage of the 163 ASCVD SNPs between shotgun and normalized capture datasets

Coverage depths of the single SNPs are presented. Other than in figure 2CXXX, the ancient Egyptian Individuals 1983 and 2509 do not show any covered target-SNPs in the captured dataset as only normalized datasets are plotted.

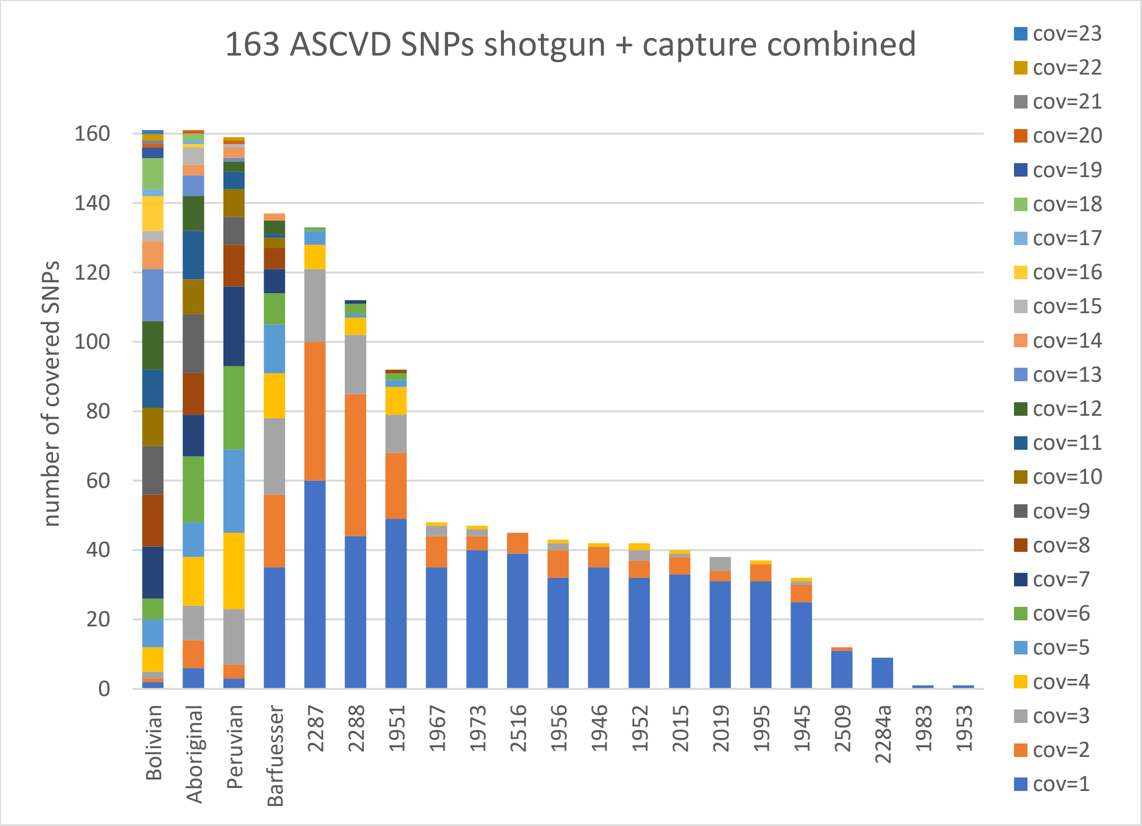

Figure S4: Coverage of all sequenced 163 ASCVD SNPs

All Shotgun and capture datasets combined.

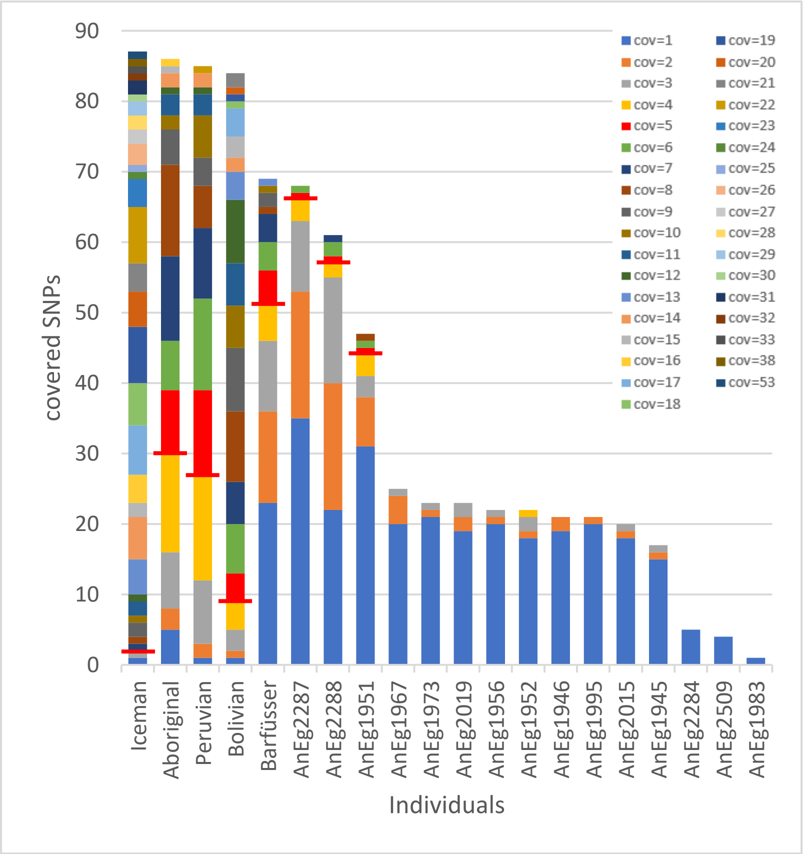

Figure S5: Coverage of the final 87 ASCVD SNPs

On the X-axis the number of the 87 ASCVD SNPs is marked. In addition, the sequencing depth of the single SNPs is shown for the different individuals. Above red lines: SNPs with a sequencing depth ≥ 5.

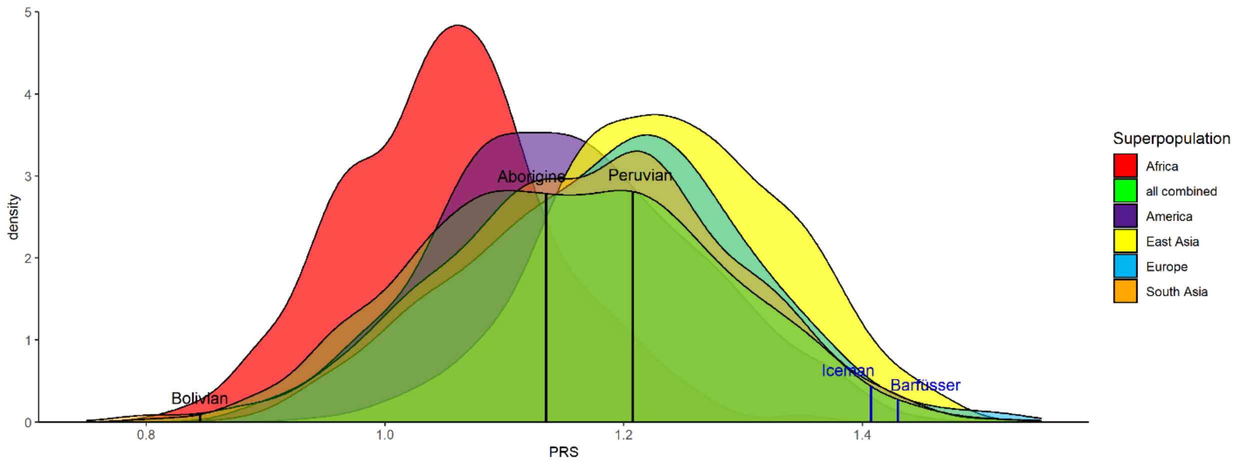

Figure S6: Density curves of PRSs with the modern individuals divided into superpopulations.

Based on the classification of the 1000 Genomes Project [8] single density curves are plotted for each superpopulation. PRSs of ancient individuals are plotted on top of the modern individuals. In blue: Individuals with calcified plaques identified on CT scans.

References:

1. Walsh S, Liu F, Wollstein A, et al. The HIrisPlex system for simultaneous prediction of hair and eye colour from DNA. *Forensic Sci Int Genet*. 2013;7(1):98-115. doi:10.1016/j.fsigen.2012.07.005

2. Hart KL, Kimura SL, Mushailov V, Budimlija ZM, Prinz M, Wurmbach E. Improved eye- and skin-color prediction based on 8 SNPs. *Croat Med J*. 2013;54(3). doi:10.3325/cmj.2013.54.248

3. Wolpin BM, Kraft P, Xu M, et al. Variant ABO blood group alleles, secretor status, and risk of pancreatic cancer: results from the pancreatic cancer cohort consortium. *Cancer Epidemiol Biomarkers Prev*. 2010;19(12):3140-3149. doi:10.1158/1055-9965.EPI-10-0751

4. Fry AE, Griffiths MJ, Auburn S, et al. Common variation in the ABO glycosyltransferase is associated with susceptibility to severe Plasmodium falciparum malaria. *Hum Mol Genet*. 2008;17(4):567. doi:10.1093/HMG/DDM331

5. Sibley E, Ahn JK. Theodore E. Woodward Award: lactase persistence SNPs in African populations regulate promoter activity in intestinal cell culture. *Trans Am Clin Climatol Assoc*. 2011;122:155-165. /pmc/articles/PMC3116366/

6. Ghirotto S, Tassi F, Barbujani G, et al. The uromodulin gene locus shows evidence of pathogen adaptation through human evolution. *J Am Soc Nephrol*. 2016;27(10):2983-2996. doi:10.1681/ASN.2015070830

7. Ralf A, van Oven M, Zhong K, Kayser M. Simultaneous analysis of hundreds of Y-chromosomal

SNPs for high-resolution paternal lineage classification using targeted semiconductor sequencing. *Hum Mutat*. 2015;36(1):151-159. doi:10.1002/humu.22713

8. Auton A, Abecasis GR, Altshuler DM, et al. A global reference for human genetic variation. *Nature*. 2015;526(7571):68-74. doi:10.1038/nature15393
